# Intraspecific variation in antibiotic resistance potential within *E. coli*

**DOI:** 10.1128/spectrum.03162-23

**Published:** 2024-04-25

**Authors:** Stacy A. Suarez, Adam C. Martiny

**Affiliations:** 1 Department of Ecology and Evolutionary Biology, University of California, Irvine, California, USA; 2 Department of Earth System Science, University of California, Irvine, California, USA; University at Albany, Albany, New York, USA

**Keywords:** antibiotic resistance, intraspecific variation, functional metagenomics, drug resistance mechanisms, drug resistance evolution

## Abstract

Intraspecific genomic diversity brings the potential for an unreported and diverse reservoir of cryptic antibiotic resistance genes in pathogens, as cryptic resistance can occur without major mutations and horizontal transmission. Here, we predicted the differences in the types of antibiotics and genes that induce cryptic and latent resistance between micro-diverse *Escherichia coli* strains. For example, we hypothesize that known resistance genes will be the culprit of latent resistance within clinical strains. We used a modified functional metagenomics method to induce expression in eight *E. coli* strains. We found a total of 66 individual genes conferring phenotypic resistance to 11 out of 16 antibiotics. A total of 14 known antibiotic resistance genes comprised 21% of total identified genes, whereas the majority (52 genes) were unclassified cryptic resistance genes. Between the eight strains, 1.2% of core orthologous genes were positive (conferred resistance in at least one strain). Sixty-four percent of positive orthologous genes conferred resistance to only one strain, demonstrating high intraspecific variability of latent resistance genes. Cryptic resistance genes comprised most resistance genes among laboratory and clinical strains as well as natural, semisynthetic, and synthetic antibiotics. Known antibiotic resistance genes primarily conferred resistance to multiple antibiotics from varying origins and within multiple strains. Hence, it is uncommon for *E. coli* to develop cross-cryptic resistance to antibiotics from multiple origins or within multiple strains. We have uncovered prospective and previously unknown resistance genes as well as antibiotics that have the potential to trigger latent antibiotic resistance in *E. coli* strains from varying origins.

**IMPORTANCE:**

Intraspecific genomic diversity may be a driving force in the emergence of adaptive antibiotic resistance. Adaptive antibiotic resistance enables sensitive bacterial cells to acquire temporary antibiotic resistance, creating an optimal window for the development of permanent mutational resistance. In this study, we investigate cryptic resistance, an adaptive resistance mechanism, and unveil novel (cryptic) antibiotic resistance genes that confer resistance when amplified within eight *E. coli* strains derived from clinical and laboratory origins. We identify the potential of cryptic resistance genes to confer cross-resistance to antibiotics from varying origins and within multiple strains. We discern antibiotic characteristics that promote latent resistance in multiple strains, considering intraspecific diversity. This study may help detect novel resistance genes and functional genes that could become responsible for cryptic resistance among diverse strains and antibiotics, thus also identifying potential novel antibiotic targets and mechanisms.

## INTRODUCTION

The rapid evolution and dissemination of resistance genes contribute to the antibiotic resistance crisis (1). To effectively mitigate this threat to human health (2, 3), it is important to identify and characterize antibiotic resistance (AR) genes as well as bacterial resistance mechanisms. Antibiotic resistance typically emerges due to acquired, intrinsic, or adaptive resistance mechanisms (4). Acquired resistance pertains to chromosomal mutations or the assimilation of genetic elements, while intrinsic resistance characterizes the innate properties of a bacterium to circumvent the impact of antibiotics. Adaptive resistance, caused by changes in gene expression, occurs in response to environmental conditions, such as antibiotic exposure. Latent resistance is a form of adaptive resistance and can occur from the activation of unknown (cryptic) AR within the cell (5–10). Thus far, few studies have considered the link between antibiotic resistance and the upregulation of cryptic AR genes among micro-diverse lineages (5–10).

Intraspecific variation, which includes the genomic diversity found within populations, can correspond to variation in a wide range of functional traits including antibiotic resistance (11). Intraspecific genomic diversity promotes the potential for an unreported and diverse reservoir of latent AR genes in pathogens because cryptic resistance can occur without major mutations or horizontal transmission. Large differences in genomic content have been shown among closely related *E. coli* strains (12). For example, one article detailed that three distinct *E. coli* strains shared about 40% of genes, and two of the three were clinical, pathogenic strains. These two clinical strains were as different from each other as they were from nonpathogenic strains. The acquisition of genomic islands encoding virulence factors led to pathogenicity in the clinical strains (12).

Functional metagenomics is used to investigate intraspecific latent antibiotic resistance. Microbiomes from humans (13, 14), sea gulls (15), soils (16, 17), rivers (18), and ocean water (19) have revealed reservoirs of diverse known and unknown latent AR genes. Functional metagenomics is an efficient and powerful technique for AR gene detection (20, 21) due to three key advantages: (i) no need for culturing microorganisms apart from the donor strain, (ii) no prior knowledge required about the resistance gene sequence, and (iii) a direct association between a genotype and a demonstrated resistance phenotype (22). Functional metagenomics uses a surrogate host to identify resistance genes, but this can confound results as phenotypic resistance in donor strains may not translate to resistance in the native genomic context.

We have developed an assay that circumvents this limitation and expresses genes in the organism of interest (10). Delineating the intraspecific potential for cryptic antibiotic resistance is important to further elucidate the emergence of antibiotic resistance. We use our method to test the hypothesis that there is a highly diverse reservoir of cryptic latent AR genes between strains of the same species that confer an AR phenotype when upregulated. We therefore predict that strain origin will affect the type of genes and antibiotics inducing resistance. For example, we expect that known resistance genes will primarily cause latent resistance within clinical strains. Additionally, we predict that cryptic resistance is more likely to occur to hydrophilic antibiotics due to the highly hydrophobic outer membrane in *E. coli* (10). Here, we use a functional metagenomics assay that induces a large increase in gene copy number to assay intraspecific variation in AR potential. We specifically ask the following questions: (i) what are the groups of orthologous genes (orthogroups) among *E. coli* strains that confer an AR phenotype when upregulated and (ii) how do strain and antibiotic origin affect which orthogroups induce latent resistance in this manner?

## MATERIALS AND METHODS

### Strains, media, and culture conditions

E. cloni (*E. coli* K-12 derivative) 10G supreme cells (Lucigen, Middleton, WI, USA), *E. coli* 40B, and *E. coli* 72 were grown in Luria–Bertani (LB) media and incubated overnight at 37°C, unless otherwise stated. Genomic DNA from the following strains was obtained from the American Type Culture Collection (ATCC): *E. coli* FDA strain Seattle 1946 (ATCC 25922), *E. coli* H10407 (ATCC 35401), *E. coli* Crooks (ATCC 8739), *E. coli* RIMD 0509952 (ATCC BAA-460), and *E. coli* AMC 198 (ATCC 11229). Genomic data including gene annotations for each ATCC strain are publicly available (23). *E. coli* 40B and *E. coli* 72 were isolated from the blood of infants with bacteremia at the Children’s Hospital Orange County with approval from IRB#120775. *E. coli* 40B presents H and O serotype markers, while *E. coli* 72 presents H serotype markers. Both strains harbor the resistance genes *fyuA*, *iucC*, *ompT*, and *sfaS*. We assessed latent antibiotic resistance in eight strains by transforming fractions of their DNA into E. cloni. The strains were chosen to represent clinical and laboratory origins. Only two laboratory strains were selected as most readily available laboratory *E. coli* strains are derived from *E. coli* K-12, resulting in low genetic variation between strains.

### Resistance profile

The minimum concentration of antibiotics needed to inhibit (MIC) the growth of 10^6^ E. cloni cells was determined for all antibiotics (Table 1), as described in (10). The listed antibiotics were tested to include a range of classes (mechanisms of action) and origins (natural, semisynthetic, or synthetic) if available.

**TABLE 1:** Total antibiotics tested and their respective properties

Biochemical property	Site of action	Class and subclass	Origin	Antibiotic	Antibiotic concentration^*a*^	EUCAST ECOFF^*b*^
Hydrophilic	Cell wall	Beta-lactam				
		Penicillins	Natural	Penicillin	64	NA
		Cephalosporins	Semisynthetic	Ampicillin	8	8
				Cephalothin	32	32
				Cefoxitin	64	16
				Cefotaxime	0.25	0.25
				Cefepime	0.125	0.125
		Monobactams	Synthetic	Aztreonam	0.25	0.25
		D-cycloserine	Natural	D-cycloserine	32	NA
Amphipathic	Cytoplasmic membrane	Polymyxins	Natural	Polymyxin B	0.5	NA
Hydrophobic	Protein synthesis	Chloramphenicol	Synthetic	Chloramphenicol	8	16
		Aminoglycosides	Natural	Gentamicin	4	2
			Semisynthetic	Amikacin	16	8
		Tetracyclines	Natural	Tetracycline	4	8
			Natural	Chlortetracycline	4	NA
			Semisynthetic	Doxycycline	4	8
	DNA synthesis	Fluoroquinolones	Synthetic	Nalidixic Acid	4	8
			Synthetic	Norfloxacin	0.125	NA
		Nitrofurans	Synthetic	Nitrofurantoin	1	64

^*a*^ The minimum concentration of antibiotics (ug/mL) needed to inhibit the growth of E. cloni cells (Lucigen). This concentration (MIC) was used to screen clones from all *E. coli* strains for cryptic antibiotic resistance.

^*b*^ Epidemiological cut-offs (ECOFFs) in ug/mL for *E. coli*, as defined by the European Committee on Antimicrobial Susceptibility Testing (EUCAST).

### Cloning and screening

The following methods were completed separately for each strain (10). Genomic DNA was extracted from E. cloni*, E. coli* 40B*,* and *E. coli* 72 cells using the Wizard Genomic DNA purification Kit (Promega Corporation, Madison, WI, USA). At least 5 micrograms of genomic DNA from each strain (including the ATCC strains) was sheared to a target size of 2 kb using a Covaris S220 Focus Acoustic Shearer (Covaris Inc., Woburn, MA, USA). Fragments of 1 to 3 kb were extracted from a 1% agarose gel using the Zymoclean Gel DNA Recovery Kit (Zymo Research, Irvine, CA, USA). DNA was treated with the NEBNext End Repair Module to create blunt ends on the fragmented DNA (New England Biolabs, Ipswich, MA, USA). The end-repaired DNA was purified using the DNA Clean and Concentrator-10 kit (Zymo Research). DNA was ligated into the pSMART-HCKan vector (accession number AF532107) and then electroporated into E. cloni cells following the instructions of the CloneSmart Blunt Cloning Kit (Lucigen). Transformed cells were recovered at 37°C for 1 hour.

To test for cryptic antibiotic resistance, 150 µL of undiluted recovered transformants was plated on LB Lennox kanamycin agar (necessary for plasmid selection) containing one of eighteen antibiotics (Table 1). After overnight incubation, resistant transformants were pooled for each antibiotic using 1–2 mL of phosphate-buffered saline (PBS). Pooled plasmid DNA was extracted from each PBS suspension (one from each resistance-positive antibiotic) using the ZR Plasmid Miniprep kit (Zymo Research) and stored at −20°C. Plasmid inserts containing latent AR genes were amplified via the polymerase chain reaction (PCR). This PCR used 25-uL reactions, including 12.5 uL of AccuStart II PCR SuperMix 2X (Quantabio), 3 uL (1.5 ng) of plasmid DNA, 4.5 uL of nuclease-free water, and 2.5 uL of SL1 and SR2 primers (Lucigen). The reaction cycle conditions follow those delineated for AccuStart II PCR SuperMix 2X (Quantabio). PCR products were purified using the QIAquick PCR Purification Kit (Qiagen) and quantified using the Invitrogen Qubit fluorimeter (Thermo Fisher Scientific). A low quantity of DNA was generated from cephalothin and polymyxin B-resistant clones, suggesting that these were not plasmid-containing colonies. Therefore, these clones were excluded from subsequent analysis and sequencing for all strains.

### Library preparation, sequencing, and analysis

For pooled plasmids (separated by strain), library preparation and sequencing were performed (10). Sequencing was done on the MinION flow cell (FLO-Min106 R9.4.1 version; Oxford Nanopore Technologies) using the MinION device (Mk1B version). Base-calling was done in real-time using MinKNOW software (Oxford Nanopore Technologies) on a local computer. Each sequencing run was carried out for about 15 hours, and the barcoded base-called reads were demultiplexed by MinKNOW during the sequencing run. Demultiplexed reads were trimmed post-sequencing using MinKNOW to remove barcodes. Trimmed reads were aligned and mapped to their respective *E. coli* reference genome using Bowtie 2 (24). Mapped reads were assembled and processed with Anvi’o (25), which provided coverage, identity, and location within the reference strain for each aligned gene.

We fit a gamma distribution to gene coverage values and selected genes that had coverage within the 95% CI as putative resistance genes. Gene coverage values were normalized by total coverage values for each resistance-positive antibiotic prior to obtaining the confidence interval. Gene identities were confirmed with NCBI BLASTx, and gene names present within the Comprehensive AR Database, CARD (26), were identified as known AR genes. Gene names not present within the CARD were designated as cryptic/unknown AR genes. Latent AR genes include known and cryptic AR genes. For each resistance-positive antibiotic, we identified the gene with the highest coverage as the most probable resistance gene when multiple genes were located within close proximity in the respective reference strain (i.e., the eight *E. coli* strains). After taking this into account, we found a total of 66 individual AR genes across all resistance-positive antibiotics from all strains.

**Fig 1:**
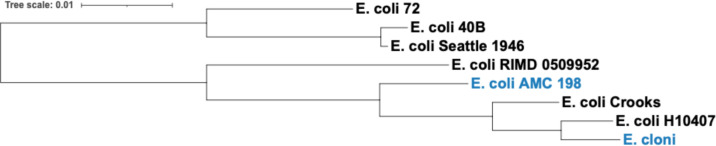
Genetic relatedness between all strains tested for cryptic antibiotic resistance. The strains in blue are laboratory strains, and those in black are clinical strains.

OrthoFinder 2.0 (27) was used to find groups of orthologous genes (orthogroups), and the Interactive Tree of Life v5 (28) was used to build the phylogenetic tree showing the genetic relatedness among all strains (Fig. 1). For heatmap hierarchical clustering of orthogroups and strains (based on positive antibiotic resistance genes), R’s “ggdendro” package was used. The “ggplot2” package was used for displaying the heatmap dendrogram clustering. To determine the correlation between the dendrograms generated based on phylogeny (Fig. 1) and resistance profile (Fig. 5), R’s “vegan” package was used to perform the Mantel test.

## RESULTS AND DISCUSSION

Through a modified functional metagenomics approach (Fig. S1), we tested for the intraspecific potential of cryptic antibiotic resistance in eight *E. coli* strains (Fig. 1). In this study, “resistance” meant that clones were able to grow at the MICs determined from the original host strain (E. cloni). We conducted a quantitative analysis of latent AR genes according to their functional categories and between strains. We examined the relation between cryptic/latent resistance and strain origin and antibiotic type. Thus, we characterized the intraspecific variation of the cryptic/latent AR potential by this gene amplification assay.

**Fig 2:**
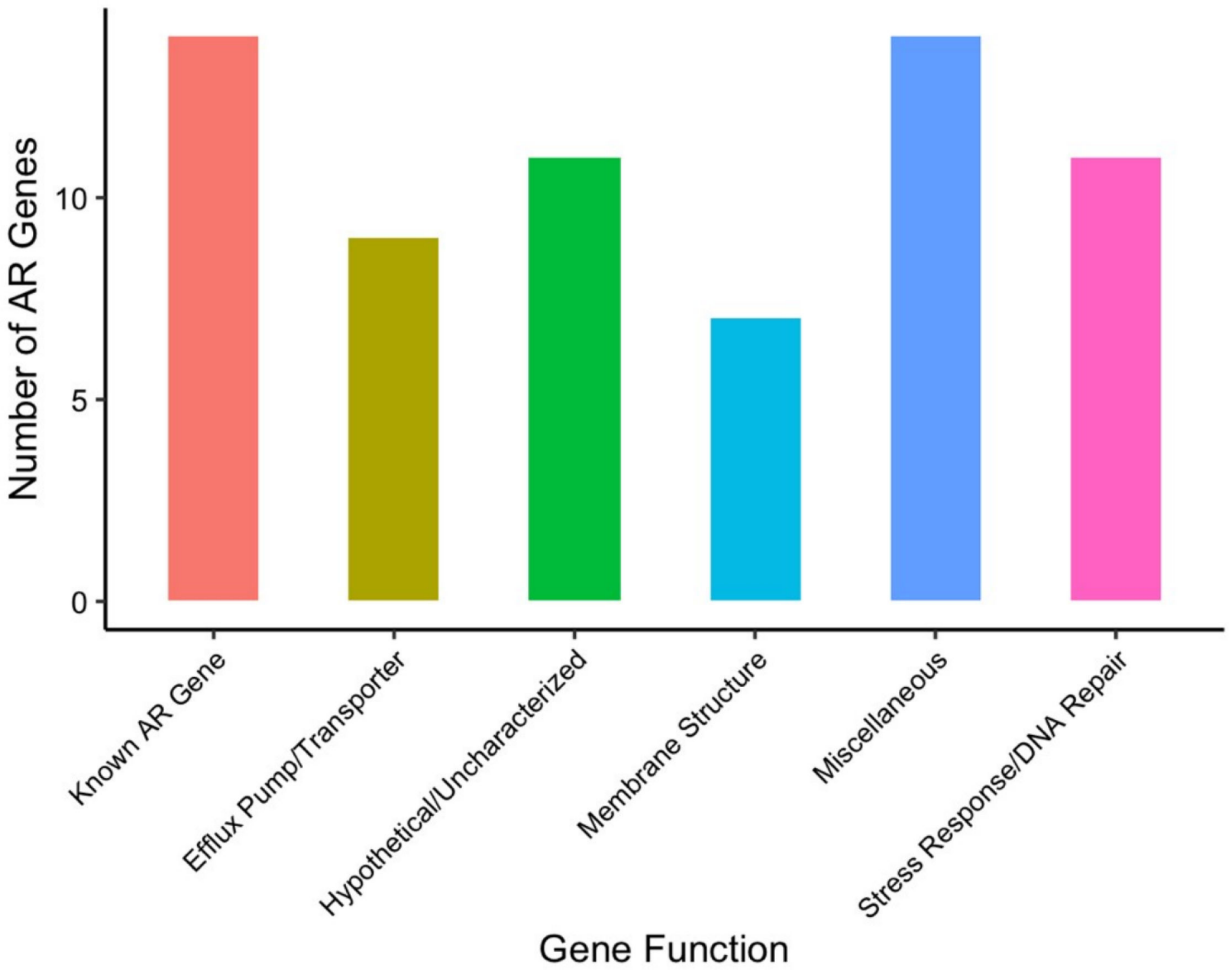
Distribution of antibiotic resistance gene functional categories conferring latent resistance at the MICs. We identified a total of 66 individual resistance orthologous genes across all resistance-positive antibiotics from all strains.

**Fig 3:**
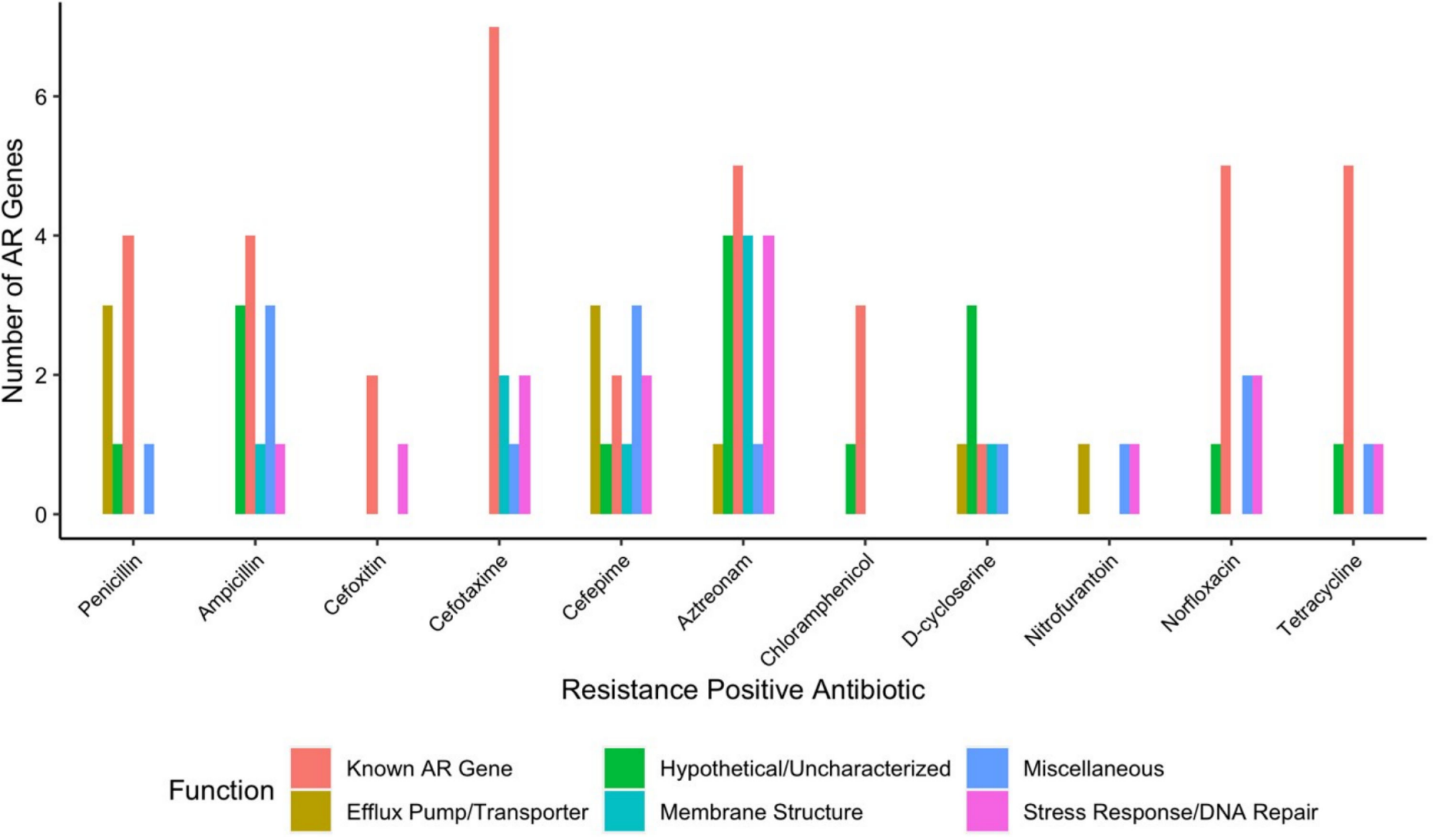
Number of antibiotic resistance genes conferring latent resistance to antibiotics at the MICs. Penicillin–aztreonam are beta-lactams.

**Fig 4:**
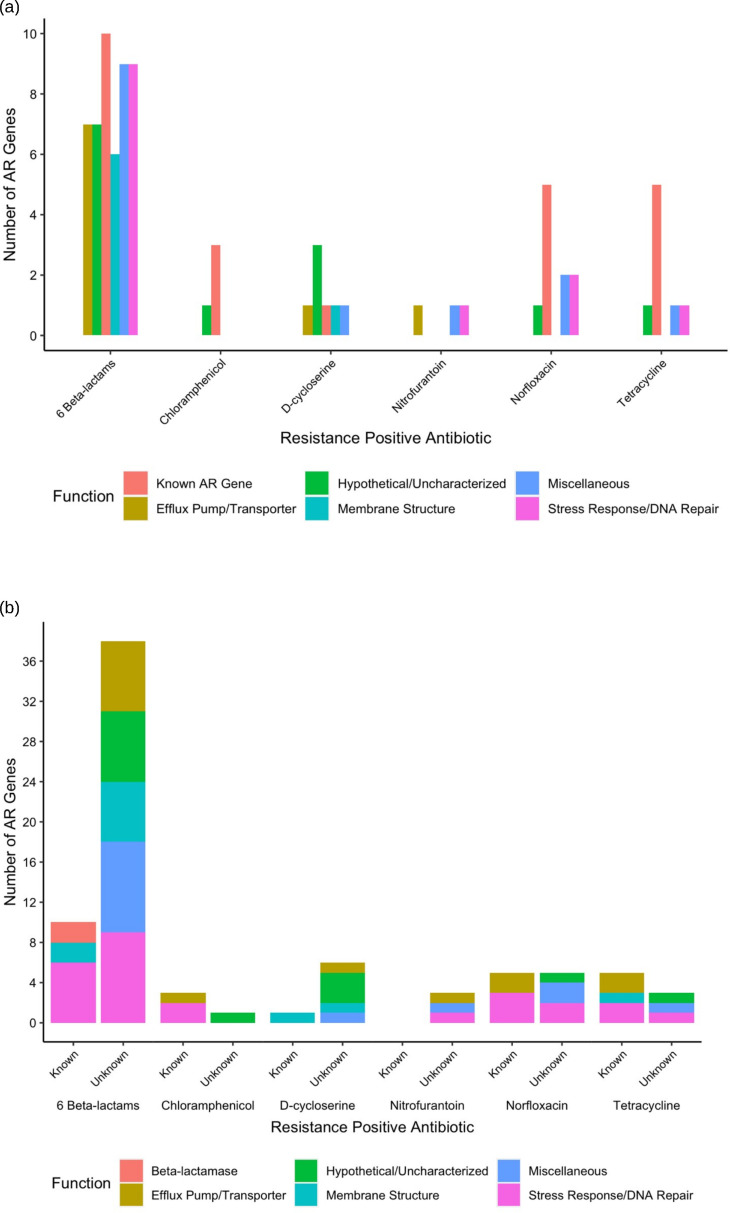
Number of antibiotic resistance genes conferring latent resistance to antibiotics at the MICs, separated by class (a) and by known/unknown AR genes (b).

We observed a wide diversity of latent AR genes. We found a total of 66 individual genes conferring latent resistance to 11 out of 16 tested antibiotics. Known resistance types (CARD-positive) comprised 21% of identified AR genes, whereas the majority (79%) of the identified AR genes were unclassified (CARD-negative) (Fig. 2). Resistance-positive antibiotics included chloramphenicol, D-cycloserine, nitrofurantoin, norfloxacin, tetracycline, and six beta-lactams (Fig. 3). Latent AR gene functions vary for each antibiotic, but known AR genes conferred resistance to the highest number of antibiotics (Fig. 3). Genes from all functional categories (known AR genes, efflux pump/transporter, hypothetical/uncharacterized, membrane structure, miscellaneous, and stress response/DNA repair) conferred resistance to the class of beta-lactam antibiotics (Fig. 4a). Hypothetical/uncharacterized, stress response/DNA repair, and miscellaneous genes conferred resistance to all antibiotic classes, except for nitrofurantoin, D-cycloserine, and chloramphenicol. Genes related to membrane structure conferred resistance to beta-lactams and D-cycloserine (Fig. 4a). This result was expected as beta-lactams and D-cycloserine are the only antibiotic classes that inhibit cell wall synthesis. Stress response/DNA repair was the most represented (54%) gene functional category for known AR genes across all antibiotic classes (Fig. 4b). Within unknown AR genes, miscellaneous genes were the most common (25%). Stress response/DNA repair was represented to be the highest (33%) across all latent genes for all antibiotic classes (Fig. 4b). Bacterial transcriptional responses to stress have presented a lack of specificity to the given stress (29), as also shown in our study with a high proportion of miscellaneous and stress response genes. Therefore, the regulated genes may constitute an integral component of a nonspecific stress response, offering the advantage of conferring cross-protection against multiple environmental conditions that may often co-occur (29). For example, a combination of antibiotics is advantageous for cases of multidrug resistant Gram-negative infections and severe pneumonia and group A streptococcal infections (30).

**Fig 5:**
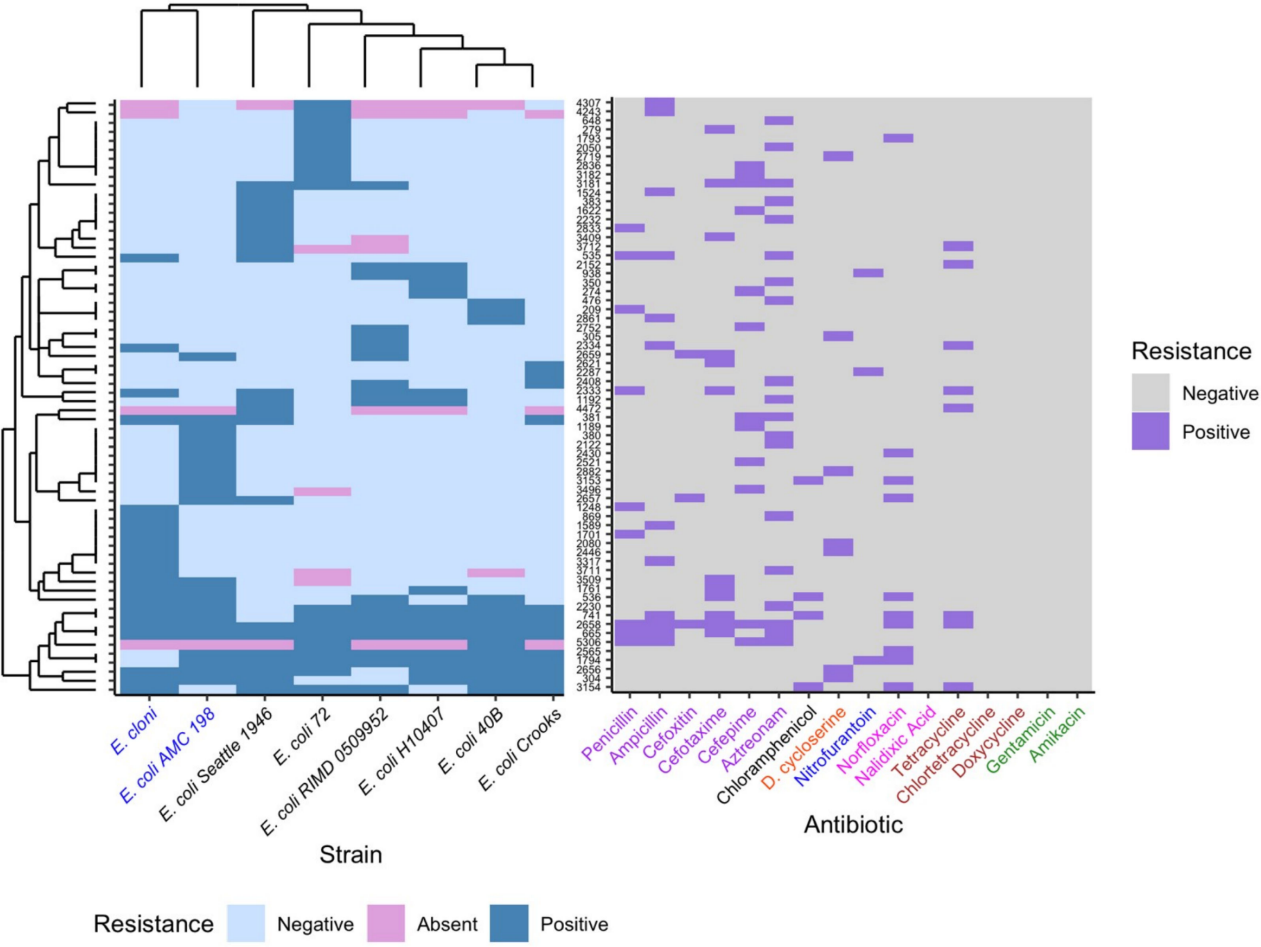
Resistance-positive shared groups of orthologous genes (left) conferring latent resistance to antibiotics (right) at the MICs. The proportion of resistance-positive orthogroups (shown) is 1.2%. The strains in blue are laboratory strains, and those in black are clinical strains. Dendrograms were built based on positive antibiotic resistance genes. Antibiotics are color-coded by class.

We next analyzed AR orthogroups shared between *E. coli* strains and the antibiotics resisted by each orthogroup (Fig. 5). Between the eight strains, a total of 35,823 genes were classified into 5,551 orthologous groups, including single-gene groups. The proportion of positive orthologs (conferred resistance in at least one strain) was 1.2% or 66 genes. Eighty-six percent of AR genes (57 genes) were shared between the eight strains, and nine AR genes were not shared within all eight strains (Fig. 5). Sixty-four percent of positive orthologs conferred resistance to only one strain, demonstrating high intraspecific variability of latent AR genes. This result is noteworthy because the majority of AR genes were shared within all strains. Multiple reasons could be the cause for high intraspecific variability, including mutation or movement of the gene within each strain. The genomic background of the gene may vary across strains. Bacterial species have shown considerable variations in genetic diversity and have displayed historical rates of recombination, horizontal gene transfer, and recurrent mutation (31–33). For example, TEM-1 beta-lactamase (orthogroup 5306), may have been horizontally transmitted in *E. coli* 72 and *E. coli* 40B as this gene conferred latent resistance while being absent in the remaining strains (Fig. 5). TEM beta-lactamases are normally transferred by horizontal gene transfer, such as plasmids as these genes can be found in many mobile genetic elements (34). Also, sampling error could be the culprit causing an incomplete screen of the genome for latent AR genes. However, with an average of 75,000 clones being tested on each antibiotic, the probability of missing a gene is very low.

Eighteen percent (12 genes) of positive orthogroups conferred resistance in at least half of all strains (0.22% of all orthologs, Fig. 5). Three of the 12 genes are unclassified AR genes: *arfB*, *recA*, and *eamA*. Alternative rescue factor A (ArfB) encodes a ribosome rescue system commonly present in bacteria (35). Although ArfB has not been directly linked to antibiotic resistance, ribosome rescue inhibitors have been suggested as potential antibiotic mechanisms (35, 36). ArfB also contributes to heat stress resistance in *Azotobacter vinelandii* (37), demonstrating how ribosome rescue mechanisms can play a role in tolerance to stressors. Even though *recA* is not a classified AR gene, it has been well-known to induce antibiotic resistance via the SOS response (38–40). EamA is an exporter classified within the drug/metabolite transporter superfamily (41). Three percent of positive orthologs conferred resistance in all eight strains. These include two known AR genes: *ampC* and *marA. ampC* is encoded on the chromosomes of many *Enterobacteriaceae* but is normally expressed at low levels (42). Mutation and plasmid mediation of *ampC* can lead to overexpression, resulting in beta-lactam resistance (42). Extremely high copy numbers are not necessary to enable rapid evolution of plasmid-encoded AR genes. A multicopy number plasmid (19 copies/cell) carrying the *blaTEM-1* gene enabled resistance to ceftazidime when compared to *E. coli* carrying the gene on the chromosome (43). The combination of an increased rate of mutations in *blaTEM-1* with an improved rate of ceftazidime hydrolysis and the amplification of these mutations led to the evolution of resistance. We uncovered a highly variable intraspecific reservoir of latent AR genes, which uncommonly develop cross-cryptic resistance within multiple strains.

We found that cryptic AR genes present a low potential of developing cross-cryptic resistance to multiple antibiotics as compared to known AR genes (Fig. 5). Positive orthologs did not confer latent resistance to nalidixic acid, chlortetracycline, doxycycline, gentamicin, or amikacin. Hence, *E. coli* strains may not have the potential to develop latent or cryptic resistance to aminoglycosides. Even though the plasmid used in this assay encodes kanamycin resistance as a selection agent, and the resistance gene *aphA1* inactivates kanamycin and neomycin (44), none of the aminoglycosides tested for latent resistance in this study. One orthogroup (*marA*, known AR gene) conferred resistance to at least half of all antibiotics. Four orthogroups of known AR genes conferred resistance to at least half of all antibiotic classes. These genes are *marA*, *soxS*, *robA*, and *mdfA*. Thus, *E. coli* strains may not be as capable of developing cross-cryptic resistance to multiple antibiotics as known AR genes conferred resistance to at least half of all antibiotics and antibiotic classes. Seventy-seven percent of positive orthogroups conferred resistance to only one antibiotic, highlighting the variability of latent AR genes and suggesting that these genes may stem from a certain gene response specific to the antibiotic. The dendrograms in Fig. 5 are generated based on the resistance profile, and the dendrogram in Fig. 1 is generated according to genetic relatedness between the strains. It is noteworthy that the two laboratory strains are clustered within the same clade when based on the resistance potential. Although the two dendrograms differ, there is phylogenetic conservatism to the antibiotic resistance potential as there is a significant relationship between the resistance profile and phylogeny (Mantel test, *P* < 0.05).

**Fig 6:**
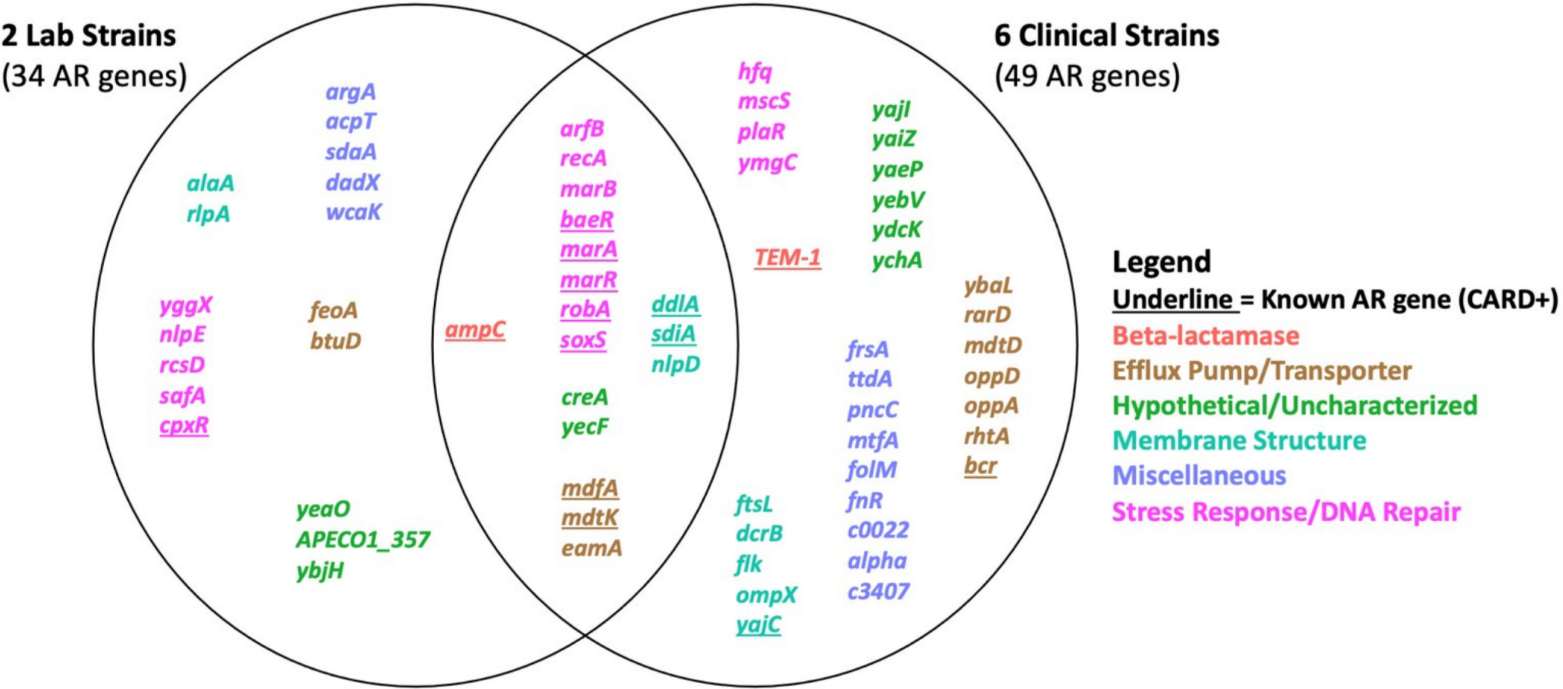
Antibiotic resistance genes shared between all strains, separated by strain origin. We identified 66 antibiotic resistance genes (shown) causing resistance at the MICs. Known antibiotic resistance genes were classified using the comprehensive antibiotic resistance database by gene name.

A vast reservoir of cryptic AR genes conferred resistance across strains from laboratory and clinical origins (Fig. 6). Sixty-eight percent and 73% of genes conferring latent resistance in laboratory and clinical strains were unclassified AR genes. However, most latent AR genes (59%) conferring cross-resistance in strains from laboratory and clinical origins were known AR genes. Known AR genes may not always be the culprit of latent resistance in clinical strains, as we predicted. The cryptic AR genes conferring resistance in strains from both origins are *arfB*, *recA*, *marB*, *creA*, *yecF*, *nlpD*, and *eamA*. MarB is part of the multiple antibiotic resistance operon, marRAB, in which *marA* and *marR* are classified AR genes (45). MarB has an unknown function, but it has been shown to increase the level of MarA. CreA has an uncharacterized function, but it is adjacent to the CreBC two-component regulatory system and *robA*, a known AR gene (46). *creA* was also shown to confer cryptic resistance to multiple beta-lactam antibiotics from varying origins (10). YecF has an uncharacterized function, but it has been shown to be upregulated in response to antibiotic exposure (10, 47). Additionally, YecF is adjacent to *sdiA*, a known AR gene (48). NlpD is involved in maintaining cell membrane permeability and integrity (49). Since *nlpD* conferred resistance to cefotaxime, this alleviates the stress on cell wall biosynthesis caused by the beta-lactam cefotaxime (50). Here, *eamA* conferred resistance to D-cycloserine in seven *E. coli* strains, and it has also been shown to confer cryptic resistance to D-cycloserine in a laboratory *E. coli* strain (10). For both origins (individually and combined), stress response genes comprised the highest number of AR genes compared to other gene functions, demonstrating the broad intraspecific latent AR potential for this gene function. Bacterial stress response mechanisms such as the general (51, 52), SOS (38–40), oxidative (53, 54), and envelope stress responses (55, 56) have been commonly shown to reduce antibiotic susceptibility. Even though known AR genes contributed to cross-resistance within distinct strains, a diversity of cryptic AR genes led to cryptic resistance among *E. coli* strains.

**Fig 7:**
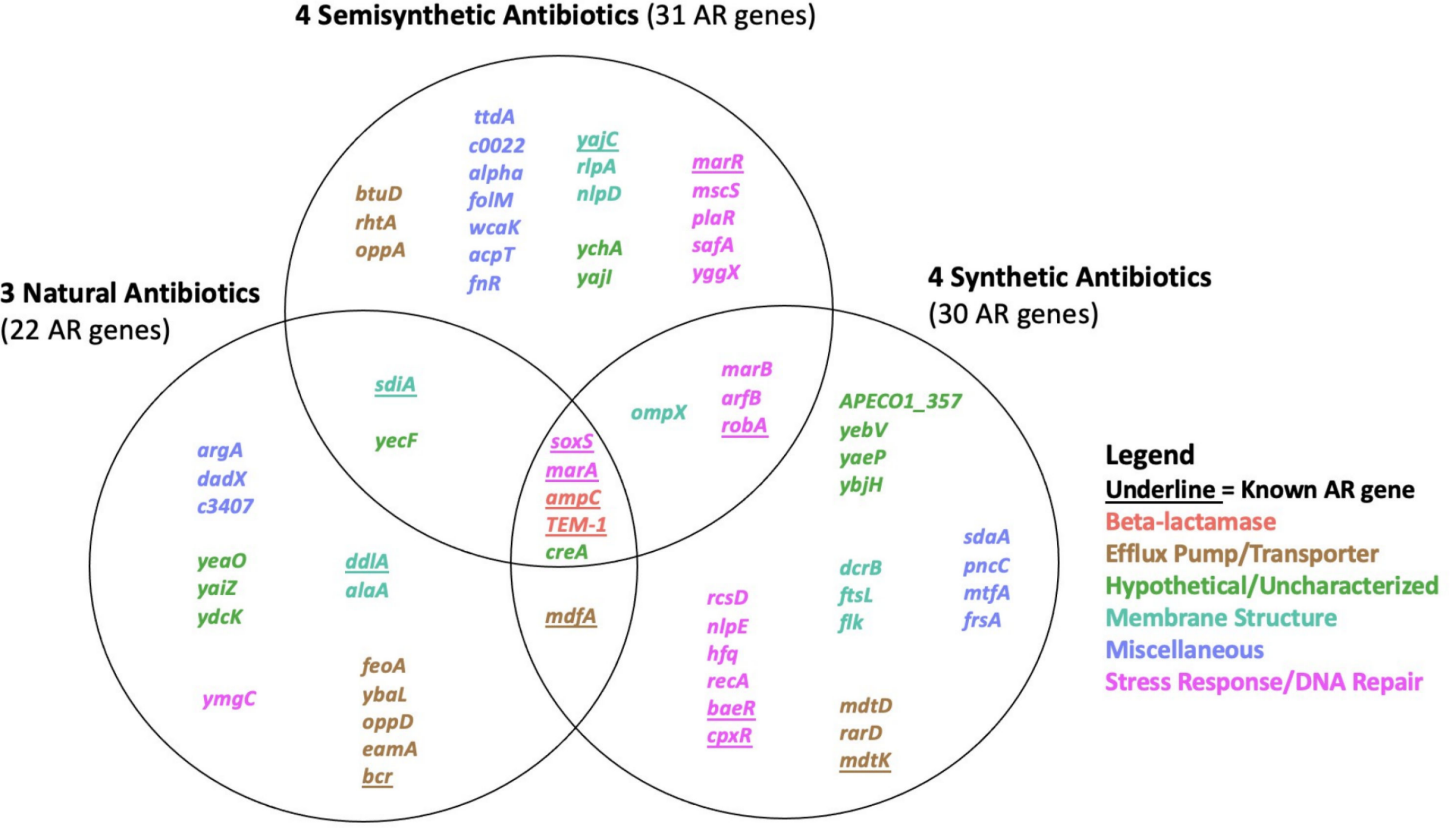
Antibiotic resistance genes shared between all strains, separated by resistance-positive antibiotic origin. We identified 66 antibiotic resistance genes (shown) causing resistance at the MICs. Known antibiotic resistance genes were classified using the comprehensive antibiotic resistance database by gene name.

We found that cross-latent resistance to multiple origins of antibiotics is driven by known AR genes (Fig. 7). Specifically, 80% of genes conferring latent resistance to all antibiotic origins (natural, semisynthetic, and synthetic) are known AR genes. The only cryptic AR gene conferring resistance to all antibiotic origins is *creA*. Even though known AR genes primarily conferred cross-latent resistance to multiple antibiotic origins, cryptic AR genes comprised the majority of natural [64%], semisynthetic [74%], and synthetic [70%] antibiotics. There were the fewest natural resistance-positive antibiotics [3], compared to semisynthetic [4] or synthetic [4] antibiotics. We had predicted that latent resistance would be most common in the presence of natural antibiotics, but this was not the case as presented in this study and previously (10). Additionally, latent resistance occurred to all hydrophilic antibiotics (Table 1), as we predicted. This occurred possibly due to the highly hydrophobic outer membrane present in Gram-negative bacteria being a barrier for hydrophilic (water-soluble) antibiotics (10). Sixty-four percent of resistance-positive antibiotics were hydrophilic and inhibited cell wall synthesis (Table 1), potentially showing a link between antibiotic mechanism of action and latent resistance. Antibiotics that inhibited the cell wall synthesis or cytoplasmic membrane also comprised most resistance-positive antibiotics when testing for latent resistance in a laboratory strain of *E. coli* (10). For semisynthetic and synthetic antibiotics, stress response/DNA repair genes comprised the highest number of AR genes compared to other gene functional categories, highlighting the significant role of this gene functional category in latent resistance. While known AR genes were the main contributors to cross-latent resistance, cryptic AR genes comprised the majority for natural, semisynthetic, and synthetic antibiotics.

### Methodological considerations

Our study demonstrates an unambiguous increase in the resistance level of *E. coli* K-12 (E. cloni) due to the presence of latent AR genes. We carefully tested the MICs by replicating the approach (LB agar plate) that was used to screen clones for cryptic antibiotic resistance. We observed how latent resistance genes, including cryptic and known AR genes, allowed *E. coli* K-12 to grow at the MICs of a variety of antibiotics. However, for many genes, the increase in resistance was moderate as 50% increased the MIC for a factor of 2 in our earlier evaluation of this approach (10). In contrast, latent AR genes increased the MIC of *E. coli* K-12 to 4× the MIC of only two resistance-positive antibiotics. However, there may be more effective latent resistance genetic systems that were not picked up as our system would not capture complex gene regulation or require multiple genes.

In terms of interpreting our data in a broader sense for antibiotic resistance, there are additional considerations. The MIC methodology used in this study was specifically tailored to align with our assay as our primary objective was to identify new latent biological resistance mechanisms. Translation for a clinical setting would require further examination of the inhibition concentrations using a clinical standard method with more clinical strains. Latent resistance was observed for nitrofurantoin, tetracycline, and chloramphenicol, which have an MIC value less than the ECOFF (57) system (Table 1). In a management context, these instances may not be labeled as antibiotic resistance, but our gene amplification assay has illustrated how they have caused a change in the resistance profile in our target strain. Through this study and previously (10), we presented how this genetic mechanism increases the resistance level in *E. coli* K-12 through a wide diversity of latent AR genes. Given the general biological similarity among *E. coli* strains, we would strongly predict that the overexpression of these latent AR genes would cause an increase in the resistance profile for other *E. coli* strains as well. We assume it is very likely that this would be seen for other *E. coli* strains, but the extent of the interaction between the genetic background and resistance profile remains unknown.

### Outlook

Functional metagenomic studies have shown that latent AR genes are a common occurrence among bacteria. However, due to the small insert size harboring the resistance gene, functional metagenomic studies have limited information about the phylogeny of the original host organism (20). This holds true even if used in conjunction with sequence-based metagenomics. Functional metagenomic studies have been used to identify resistance genes from certain environments but rarely from strains with distinct origins against a comprehensive panel of antibiotics. Additionally, functional metagenomic studies utilize a surrogate host. We have addressed these limitations to better comprehend the intraspecific potential for latent and cryptic antibiotic resistance. Intraspecific genomic diversity may be a driving force in the emergence of antibiotic resistance. By utilizing this platform, we aim to gain an improved understanding of the antibiotic characteristics that promote latent resistance in multiple strains, while considering intraspecific diversity. This platform offers the potential to uncover genes and functional gene categories that could become responsible for inducing cross-latent resistance to varying antibiotics within diverse strains. Thus, this study may prove valuable in the identification of novel antibiotic targets and mechanisms.

## Data Availability

Genomic data including gene annotations for each ATCC strain are publicly available within the ATCC Genome Portal (23). The following ATCC strains were used in this study: *E. coli* FDA strain Seattle 1946 (ATCC 25922), *E. coli* H10407 (ATCC 35401), *E. coli* Crooks (ATCC 8739), *E. coli* RIMD 0509952 (ATCC BAA-460), and *E. coli* AMC 198 (ATCC 11229). Genomic data for *E. coli* 40B and *E. coli* 72 are openly available in NCBI with accession numbers JBBBJU000000000 and JBBBJV000000000, respectively.

## SUPPLEMENTAL MATERIAL

Supplemental Figure spectrum.03162-23-s0001.eps Fig. S1.

ASM does not own the copyrights to Supplemental Material that may be linked to, or accessed through, an article. The authors have granted ASM a non-exclusive, world-wide license to publish the Supplemental Material files. Please contact the corresponding author directly for reuse.

## References

[1] Levy SB, Marshall B. 2004. Antibacterial resistance worldwide: causes, challenges and responses. Nat Med 10:S122–S129. doi:10.1038/nm1145 15577930

[2] US CDC. 2019. Antibiotic resistance threats in the United States. Centers Dis Control Prev:1–113. doi:10.15620/cdc:82532

[3] Ventola CL. 2015. The antibiotic resistance crisis: part 1: causes and threats. P T 40:277–283. 25859123 PMC4378521

[4] Fernández L, Hancock REW. 2012. Adaptive and mutational resistance: role of porins and efflux pumps in drug resistance. Clin Microbiol Rev 25:661–681. doi:10.1128/CMR.00043-12 23034325 PMC3485749

[5] Palmer AC, Chait R, Kishony R. 2018. Nonoptimal gene expression creates latent potential for antibiotic resistance. Mol Biol Evol 35:2669–2684. doi:10.1093/molbev/msy163 30169679 PMC6231494

[6] Struble JM, Gill RT. 2009. Genome-scale identification method applied to find cryptic aminoglycoside resistance genes in Pseudomonas aeruginosa. PLoS One 4:e6576. doi:10.1371/journal.pone.0006576 19907650 PMC2771283

[7] Soo VWC, Hanson-Manful P, Patrick WM. 2011. Artificial gene amplification reveals an abundance of promiscuous resistance determinants in Escherichia coli. Proc Natl Acad Sci U S A 108:1484–1489. doi:10.1073/pnas.1012108108 21173244 PMC3029738

[8] Erickson KE, Otoupal PB, Chatterjee A. 2017. Transcriptome-level signatures in gene expression and gene expression variability during bacterial adaptive evolution. mSphere 2:e00009-17. doi:10.1128/mSphere.00009-17 28217741 PMC5311112

[9] Salipante SJ, Barlow M, Hall BG. 2003. GeneHunter, a transposon tool for identification and isolation of cryptic antibiotic resistance genes. Antimicrob Agents Chemother 47:3840–3845. doi:10.1128/AAC.47.12.3840-3845.2003 14638492 PMC296228

[10] Suarez SA, Martiny AC. 2021. Gene amplification uncovers large previously unrecognized cryptic antibiotic resistance potential in E. coli. Microbiol Spectr 9:e0028921. doi:10.1128/Spectrum.00289-21 34756069 PMC8579933

[11] Schlatter DC, Kinkel LL. 2014. Global biogeography of Streptomyces antibiotic inhibition, resistance, and resource use. FEMS Microbiol Ecol 88:386–397. doi:10.1111/1574-6941.12307 24580017

[12] Welch RA, Burland V, Plunkett G, Redford P, Roesch P, Rasko D, Buckles EL, Liou S-R, Boutin A, Hackett J, Stroud D, Mayhew GF, Rose DJ, Zhou S, Schwartz DC, Perna NT, Mobley HLT, Donnenberg MS, Blattner FR. 2002. Extensive mosaic structure revealed by the complete genome sequence of uropathogenic Escherichia coli. Proc Natl Acad Sci U S A 99:17020–17024. doi:10.1073/pnas.252529799 12471157 PMC139262

[13] Sommer MOA, Dantas G, Church GM. 2009. Functional characterization of the antibiotic resistance reservoir in the human microflora. Science 325:1128–1131. doi:10.1126/science.1176950 19713526 PMC4720503

[14] Apjok G, Számel M, Christodoulou C, Seregi V, Vásárhelyi BM, Stirling T, Eszenyi B, Sári T, Vidovics F, Nagrand E, et al.. 2023. Characterization of antibiotic resistomes by reprogrammed bacteriophage-enabled functional metagenomics in clinical strains. Nat Microbiol 8:410–423. doi:10.1038/s41564-023-01320-2 36759752 PMC9981461

[15] Martiny AC, Martiny JBH, Weihe C, Field A, Ellis JC. 2011. Functional metagenomics reveals previously unrecognized diversity of antibiotic resistance genes in gulls. Front Microbiol 2:238. doi:10.3389/fmicb.2011.00238 22347872 PMC3275322

[16] Su JQ, Wei B, Xu CY, Qiao M, Zhu YG. 2014. Functional metagenomic characterization of antibiotic resistance genes in agricultural soils from China. Environ Int 65:9–15. doi:10.1016/j.envint.2013.12.010 24412260

[17] Allen HK, Moe LA, Rodbumrer J, Gaarder A, Handelsman J. 2009. Functional metagenomics reveals diverse β-lactamases in a remote Alaskan soil. ISME J 3:243–251. doi:10.1038/ismej.2008.86 18843302

[18] Amos GCA, Zhang L, Hawkey PM, Gaze WH, Wellington EM. 2014. Functional metagenomic analysis reveals rivers are a reservoir for diverse antibiotic resistance genes. Vet Microbiol 171:441–447. doi:10.1016/j.vetmic.2014.02.017 24636906

[19] Hatosy SM, Martiny AC. 2015. The ocean as a global reservoir of antibiotic resistance genes. Appl Environ Microbiol 81:7593–7599. doi:10.1128/AEM.00736-15 26296734 PMC4592852

[20] Mullany P. 2014. Functional metagenomics for the investigation of antibiotic resistance. Virulence 5:443–447. doi:10.4161/viru.28196 24556726 PMC3979872

[21] Dos Santos DFK, Istvan P, Quirino BF, Kruger RH. 2017. Functional metagenomics as a tool for identification of new antibiotic resistance genes from natural environments. Microb Ecol 73:479–491. doi:10.1007/s00248-016-0866-x 27709246

[22] Pehrsson EC, Forsberg KJ, Gibson MK, Ahmadi S, Dantas G. 2013. Novel resistance functions uncovered using functional metagenomic investigations of resistance reservoirs. Front Microbiol 4:145. doi:10.3389/fmicb.2013.00145 23760651 PMC3675766

[23] Benton B, King S, Greenfield SR, Puthuveetil N, Reese AL, Duncan J, Marlow R, Tabron C, Pierola AE, Yarmosh DA, Combs PF, Riojas MA, Bagnoli J, Jacobs JL. 2021. The ATCC genome portal: microbial genome reference standards with data provenance. Microbiol Resour Announc 10:e0081821. doi:10.1128/MRA.00818-21 34817215 PMC8612085

[24] Langmead B, Salzberg SL. 2012. Fast gapped-read alignment with Bowtie 2. Nat Methods 9:357–359. doi:10.1038/nmeth.1923 22388286 PMC3322381

[25] Eren AM, Esen ÖC, Quince C, Vineis JH, Morrison HG, Sogin ML, Delmont TO. 2015. Anvi'o: an advanced analysis and visualization platform for 'omics data. PeerJ 3:e1319. doi:10.7717/peerj.1319 26500826 PMC4614810

[26] Alcock BP, Raphenya AR, Lau TTY, Tsang KK, Bouchard M, Edalatmand A, Huynh W, Nguyen A-LV, Cheng AA, Liu S, et al.. 2020. CARD 2020: antibiotic resistome surveillance with the comprehensive antibiotic resistance database. Nucleic Acids Res 48:D517–D525. doi:10.1093/nar/gkz935 31665441 PMC7145624

[27] Emms DM, Kelly S. 2019. OrthoFinder: phylogenetic orthology inference for comparative genomics. Genome Biol 20:238. doi:10.1186/s13059-019-1832-y 31727128 PMC6857279

[28] Letunic I, Bork P. 2021. Interactive tree of life (iTOL) v5: an online tool for phylogenetic tree display and annotation. Nucleic Acids Res 49:W293–W296. doi:10.1093/nar/gkab301 33885785 PMC8265157

[29] López-Maury L, Marguerat S, Bähler J. 2008. Tuning gene expression to changing environments: from rapid responses to evolutionary adaptation. Nat Rev Genet 9:583–593. doi:10.1038/nrg2398 18591982

[30] York JA, Varadarajan M, Barlow G. 2020. When are combinations of antibiotics clinically useful? Br J Hosp Med (Lond) 81:1–9. doi:10.12968/hmed.2019.0348 32097069

[31] Read TD, Massey RC. 2014. Characterizing the genetic basis of bacterial phenotypes using genome-wide association studies: a new direction for bacteriology. Genome Med 6:109. doi:10.1186/s13073-014-0109-z 25593593 PMC4295408

[32] Hanage WP. 2016. Not so simple after all: bacteria, their population genetics, and recombination. Cold Spring Harb Perspect Biol 8:a018069. doi:10.1101/cshperspect.a018069 27091940 PMC4930924

[33] Martínez-Carranza E, Barajas H, Alcaraz L-D, Servín-González L, Ponce-Soto G-Y, Soberón-Chávez G. 2018. Variability of bacterial essential genes among closely related bacteria: the case of Escherichia coli. Front Microbiol 9:1059. doi:10.3389/fmicb.2018.01059 29910775 PMC5992433

[34] Xiao L, Wang X, Kong N, Zhang L, Cao M, Sun M, Wei Q, Liu W. 2019. Characterization of beta-lactamases in bloodstream-infection Escherichia coli: dissemination of bla_ADC–162_ and bla_CMY–2_ among bacteria via an IncF plasmid. Front Microbiol 10:2175. doi:10.3389/fmicb.2019.02175 31632358 PMC6781614

[35] Srinivas P, Goralski TDP, Keiler KC, Dunham CM. 2019. Alternative mechanisms of ribosome stalling rescue in the Gram‐negative bacterium Francisella tularensis. FASEB J 33:628. doi:10.1096/fasebj.2019.33.1_supplement.628.3

[36] Huang Y, Alumasa JN, Callaghan LT, Baugh RS, Rae CD, Keiler KC, McGillivray SM. 2019. A small-molecule inhibitor of trans-translation synergistically interacts with cathelicidin antimicrobial peptides to impair survival of Staphylococcus aureus. Antimicrob Agents Chemother 63:e02362-18. doi:10.1128/AAC.02362-18 30917982 PMC6437501

[37] Moreno S, Muriel-Millán LF, Rodríguez-Martínez K, Ortíz-Vasco C, Bedoya-Pérez LP, Espín G. 2022. The ribosome rescue pathways SsrA-SmpB, ArfA, and ArfB mediate tolerance to heat and antibiotic stresses in Azotobacter vinelandii. FEMS Microbiol Lett 369:fnac104. doi:10.1093/femsle/fnac104 36368695

[38] Smith PA, Romesberg FE. 2007. Combating bacteria and drug resistance by inhibiting mechanisms of persistence and adaptation. Nat Chem Biol 3:549–556. doi:10.1038/nchembio.2007.27 17710101

[39] Bunnell BE, Escobar JF, Bair KL, Sutton MD, Crane JK. 2017. Zinc blocks SOS-induced antibiotic resistance via inhibition of RecA in Escherichia coli. PLoS One 12:e0178303. doi:10.1371/journal.pone.0178303 28542496 PMC5440055

[40] Dawan J, Ahn J. 2022. Bacterial stress responses as potential targets in overcoming antibiotic resistance. Microorganisms 10:1385. doi:10.3390/microorganisms10071385 35889104 PMC9322497

[41] Jack DL, Yang NM, Saier MH. 2001. The drug/metabolite transporter superfamily. Eur J Biochem 268:3620–3639. doi:10.1046/j.1432-1327.2001.02265.x 11432728

[42] Jacoby GA. 2009. AmpC β-lactamases. Clin Microbiol Rev 22:161–182. doi:10.1128/CMR.00036-08 19136439 PMC2620637

[43] San Millan A, Escudero JA, Gifford DR, Mazel D, MacLean RC. 2016. Multicopy plasmids potentiate the evolution of antibiotic resistance in bacteria. Nat Ecol Evol 1:10. doi:10.1038/s41559-016-0010 28812563

[44] Harmer CJ, Lebreton F, Stam J, McGann PT, Hall RM. 2022. Mechanisms of IS 26-mediated amplification of the aphA1 gene leading to tobramycin resistance in an Acinetobacter baumannii isolate. Microbiol Spectr 10:e0228722. doi:10.1128/spectrum.02287-22 36073931 PMC9602291

[45] Duval V, Lister IM. 2013. MarA, SoxS and Rob of Escherichia coli - global regulators of multidrug resistance, virulence and stress response. Int J Biotechnol Wellness Ind 2:101–124. doi:10.6000/1927-3037.2013.02.03.2 24860636 PMC4031692

[46] Avison MB, Horton RE, Walsh TR, Bennett PM. 2001. Escherichia coli CreBC is a global regulator of gene expression that responds to growth in minimal media. J Biol Chem 276:26955–26961. doi:10.1074/jbc.M011186200 11350954

[47] Bie L, Zhang M, Wang J, Fang M, Li L, Xu H, Wang M. 2023. Comparative analysis of transcriptomic response of Escherichia coli K-12 MG1655 to nine representative classes of antibiotics. Microbiol Spectr 11:e0031723. doi:10.1128/spectrum.00317-23 36853057 PMC10100721

[48] Wei Y, Lee J-M, Smulski DR, LaRossa RA. 2001. Global impact of sdiA amplification revealed by comprehensive gene expression profiling of Escherichia coli. J Bacteriol 183:2265–2272. doi:10.1128/JB.183.7.2265-2272.2001 11244066 PMC95133

[49] Tsang MJ, Yakhnina AA, Bernhardt TG. 2017. NlpD links cell wall remodeling and outer membrane invagination during cytokinesis in Escherichia coli. PLoS Genet 13:e1006888. doi:10.1371/journal.pgen.1006888 28708841 PMC5533458

[50] Nikolaidis I, Favini-Stabile S, Dessen A. 2014. Resistance to antibiotics targeted to the bacterial cell wall. Protein Sci 23:243–259. doi:10.1002/pro.2414 24375653 PMC3945833

[51] Tahmasebi H, Dehbashi S, Arabestani MR. 2021. Antibiotic resistance alters through iron-regulating sigma factors during the interaction of Staphylococcus aureus and Pseudomonas aeruginosa. Sci Rep 11:18509. doi:10.1038/s41598-021-98017-5 34531485 PMC8445946

[52] Cremanns M, Lange F, Gatermann SG, Pfennigwerth N. 2022. Effect of sigma E on carbapenem resistance in OXA-48-producing Klebsiella pneumoniae. J Antimicrob Chemother 77:1578–1585. doi:10.1093/jac/dkac078 35265984

[53] Dwyer DJ, Kohanski MA, Collins JJ. 2009. Role of reactive oxygen species in antibiotic action and resistance. Curr Opin Microbiol 12:482–489. doi:10.1016/j.mib.2009.06.018 19647477 PMC2761529

[54] Poole K. 2012. Bacterial stress responses as determinants of antimicrobial resistance. J Antimicrob Chemother 67:2069–2089. doi:10.1093/jac/dks196 22618862

[55] Nishino K, Yamasaki S, Hayashi-Nishino M, Yamaguchi A. 2010. Effect of NlpE overproduction on multidrug resistance in Escherichia coli. Antimicrob Agents Chemother 54:2239–2243. doi:10.1128/AAC.01677-09 20211889 PMC2863614

[56] Guest RL, Raivio TL. 2016. Role of the Gram-negative envelope stress response in the presence of antimicrobial agents. Trends Microbiol 24:377–390. doi:10.1016/j.tim.2016.03.001 27068053

[57] Kahlmeter G, Brown DFJ, Goldstein FW, MacGowan AP, Mouton JW, Osterlund A, Rodloff A, Steinbakk M, Urbaskova P, Vatopoulos A. 2003. European harmonization of MIC breakpoints for antimicrobial susceptibility testing of bacteria. J Antimicrob Chemother 52:145–148. doi:10.1093/jac/dkg312 12837738

